# Daily Oxygen/O_3_ Treatment Reduces Muscular Fatigue and Improves Cardiac Performance in Rats Subjected to Prolonged High Intensity Physical Exercise

**DOI:** 10.1155/2015/190640

**Published:** 2015-07-21

**Authors:** C. Di Filippo, M. C. Trotta, R. Maisto, D. Siniscalco, M. Luongo, L. Mascolo, R. Alfano, M. Accardo, C. Rossi, F. Ferraraccio, M. D'Amico

**Affiliations:** ^1^Department of Experimental Medicine, Section of Pharmacology “L. Donatelli”, Second University of Naples, 80138 Naples, Italy; ^2^Department of Anaesthetic, Surgical and Emergency Sciences, Second University of Naples, 80138 Naples, Italy; ^3^Department of Physical and Mental Health and Preventive Medicine, Second University of Naples, 80138 Naples, Italy; ^4^Department Magrassi-Lanzara, Second University of Naples, 80138 Naples, Italy

## Abstract

Rats receiving daily intraperitoneal administration of O_2_ and running on a treadmill covered an average distance of 482.8 ± 21.8 m/week as calculated during 5-week observation. This distance was increased in rats receiving daily intraperitoneal administration of an oxygen/O_3_ mixture at a dose of 100; 150; and 300 *μ*g/kg with the maximum increase being +34.5% at 300 *μ*g/kg and still present after stopping the administration of oxygen/O_3_. Oxygen/O_3_ decreased the mean arterial blood pressure (−13%), the heart rate (−6%), the gastrocnemius and cardiac hypertrophy, and fibrosis and reduced by 49% the left ventricular mass and relative wall thickness measurements. Systolic and diastolic functions were improved in exercised oxygen/O_3_ rats compared to O_2_ rats. Oxygen/O_3_ treatment led to higher MPI index starting from the dose of 150 *μ*g/kg (*p* < 0.05) and more effective (+14%) at a dose of 300 *μ*g/kg oxygen/O_3_. Oxygen/O_3_ dose-dependently increased the expression of the antioxidant enzymes Mn-SOD and GPx1 and of eNOS compared to the exercised O_2_ rats. The same doses resulted in decrease of LDH levels, CPK, TnI, and nitrotyrosine concentration in the heart and gastrocnemius tissues, arguing a beneficial effect of the ozone molecule against the fatigue induced by a prolonged high intensity exercise.

## 1. Introduction

Exercise intensity has emerged as an important factor conditioning the onset of cardiovascular adaptations. Interestingly, Kemi et al. [1] showed that the effects of regular exercise training are related to different mechanisms depending on the exercise intensity: while the endothelium-dependent effects are related to lower intensity, myocardial adaptations need high intensity training over several weeks. Pluim et al. [2] reported that healthy individuals practicing regular high intensity physical activity for 5 to 6 hours per week show compensatory myocardial hypertrophy as cardiac adaptations. Acute aerobic exercise, however, results in increased free radical production [3–5], since increased exercise intensity and duration are related to lower antioxidant defenses, potentially resulting in oxidative damage to surrounding tissues [6]. Therefore, although exercise training seems to play a pivotal role of in maintaining good health, the evidence is not conclusive.

The ozone (O_3_) is a molecule made of three atoms of oxygen, characterized by a cyclic structure, dynamically unstable due to the presence of mesomeric states [7] and able to oxidize organic compounds [8]. Although the ozone shows toxic effects on the respiratory tract due to its interaction with other air pollutants [9], researchers believe that this gas has also therapeutic effects when administered in precise therapeutic doses [10–12]. These beneficial effects of oxygen/ozone mixture have been documented and are due to stimulation of the antioxidant and immune responses [10]. Oxygen/ozone therapy, therefore, is used as a complementary treatment of many diseases, including conditions characterized by high oxidative stress as chronic fatigue syndrome (CFS) [13], hypoxic state, and ischemic syndromes, in which by increasing the levels of eNOS ozone rends physiological levels of nitric oxide and thus protection from damage [14]. The aim of our study was to assess in rats whether the oxygen/ozone therapy can counteract the high muscular and cardiovascular stress arose during a prolonged high intensity exercise, and this improves the cardiovascular function and the resistance to the fatigue.

## 2. Materials and Methods

### 2.1. Animals

Healthy male Sprague Dawley rats purchased from Harlan (San Pietro al Natisone, Udine, Italy), of four–six months and weighing about 180 ± 10 g, were fed on a standard chow pellet diet and water ad libitum and were housed in individuals cage with controlled temperature (21–23°C), lighting (12-12 h light-dark cycle), and humidity (55–60%). All experimental procedures were approved by Animal Care Ethical Committee of the Naples University in accordance with Italian (Decree 116/92) and European Community (E.C. L358/1 18/12/86) Guidelines on the use and protection of laboratory animals.

40 Sprague Dawley rats were divided into 8 groups, each consisting of 5 animals. 20 rats were used in a setting of experiments lasting 11 weeks and 20 rats were used in a setting of experiments lasting 6 weeks. In each setting there were 3 groups of rats subjected to a prolonged high intensity exercise as described by Lana et al. [15] and receiving a daily intraperitoneal dose of oxygen/O_3_, one hour before the exercise training, for 5 weeks, starting from the second week of the experiment. Another group of 5 rats was subjected to the same exercise protocol but received a daily intraperitoneal injection of O_2_, serving as control. In each setting the groups were exercise training (ET) + O_2_ (control); ET + O_2_/O_3_ 100 *μ*g/kg i.p.; ET + O_2_/O_3_ 150 *μ*g/kg i.p.; and ET + O_2_/O_3_ 300 *μ*g/kg i.p.

### 2.2. Ozone Preparation

Ozone was generated with ozonator equipment from medical grade oxygen and three different volumes (1; 1.5; and 3 mL) of an oxygen/O_3_ mixture equivalent to 100; 150; and 300 *μ*g/kg i.p. were used according to our previous work [14]. The ozone concentration was measured using a UV spectrophotometer at 254 nm and it was administered immediately upon generation, an hour before the exercise training. A volume of 1; 1.5; and 3 mL of O_2_ was used as control.

### 2.3. Prolonged High Intensity Physical Exercise

The prolonged high intensity physical exercise was based on the protocol described by Lana et al. [15]; it lasted 11 weeks and was performed 5 times per week. The mean distance covered daily by the animals in each group was recorded and was averaged for all the 5 days of the week every single week and expressed as m/week ± s.e.m. In the first week the animals were acclimated to running on a treadmill for 15 minutes, 5 m/min, with 0% of inclination. After this, and until the end of the sixth week, the duration, velocity, and treadmill inclination were gradually increased until 75 minutes of duration, 25 m/min velocity, and 15% of inclination and maintained stable for an adjunctive period of 5 weeks. This protocol ensures a maximal oxygen consumption of 70% for the rats [15].

### 2.4. Physical and Hemodynamic Measurements

At the end of the exercise training protocol, the rats were anesthetized with an intramuscular injection of ketamine hydrochloride (100 mg/kg) and medetomidine (0.25 mg/kg) to carry out physical and transthoracic echocardiography determinations. Left cardiac morphology and function were evaluated using a noninvasive transthoracic M-Mode echocardiography (VisualSonics Vevo 770 imaging system with a RMV710B; Toronto, ON, Canada). Transthoracic echocardiographic determinations were performed in all 20 animals in the lateral decubitus position. The evaluation of morphometric parameters (left ventricular mass (LV mass) and relative wall thickness (RWT)), systolic function (expressed by ejection fraction (EF), fractional shortening (FS), and velocity of circumferential fiber shortening (VCF)), and diastolic function (evaluated by absolute LV isovolumetric relaxation time (IVRT), ratio of maximal early diastolic peak velocity (*E*) and late peak velocity (*A*) of mitral flow (*E*/*A* ratio)) was carried out according to VisualSonics Vevo 770 Protocol-Based Measurements and Calculations. The global index was quantified by the myocardial performance index. Mean arterial blood pressure (MAP) and heart rate (HR) were obtained by connecting the animal to a Letica system (LE 5000, Barcelona, Spain) and to a Hellige Cardioline at a speed of 50 cm/sec.

After sacrificing the animals, hearts and gastrocnemius were removed: a half-part was immersed in buffered formalin for immunohistochemical analysis, while the other half was immersed in liquid nitrogen and then stored at −20°C for carry out biochemical investigations.

### 2.5. Hematoxylin/Eosin Staining and Immunohistochemistry

Cardiac and muscular tissues were partly stained with hematoxylin and eosin and partly paraffin-embedded for immunohistochemistry. The latter ones were cut in 5 *μ*m serial section and treated with a xylene substitute (Hemo-De; Fisher Scientific) in order to remove the paraffin, and tissue sections were rehydrated with ethanol gradient washes. Tissue sections were quenched sequentially in 3% hydrogen peroxide aqueous solution and blocked with PBS 6% nonfat dry milk (Bio-Rad, Milan, Italy) for 1 h at room temperature. Sections were then incubated with specific anti-vimentin antibody. Sections were washed with PBS and incubated with secondary antibodies. Specific labeling was detected with a biotin-conjugated goat anti-rabbit IgG and avidin-biotin peroxidase complex (DBA, Milan, Italy). Immunostaining was analyzed by an expert pathologist (intraobserver variability 6%). Image program Leica IM500 and statistics program Leica QWIN were used to measure and automatically calculate the antigenic expression. Four distinct preparations for each heart and gastrocnemius sample were done and 22 fields of view were analyzed in each preparation for a total area of 1.42631*e* + 0.002 *μ*m^2^ for ×400 magnification.

### 2.6. Western Blotting Assay

Total proteins into tissues were determined by using the Bio-Rad protein assay (Bio-Rad Laboratories, Milan, Italy) on homogenate in a solution of 0.5% hexadecyl-trimethyl-ammonium bromide dissolved in 10 mM potassium phosphate buffer (pH 7) and centrifuged for 30 min at 4,000 ×g at 4°C. After gel electrophoresis in an 8% PAGE separation, the protein samples were electrotransferred onto a PVDF membrane. Blots were blocked with 5% nonfat dry milk for 1 h at room temperature and then incubated overnight with primary specific antibodies, followed by incubation with a horseradish peroxidase-conjugated secondary antibody for 1 h at room temperature. The signal was expressed as densitometric units (DU). Then, Western blots were performed to evaluate the levels in heart and gastrocnemius samples of manganese superoxide dismutase (Mn-SOD), glutathione peroxidase 1 (GPx1), lactate dehydrogenase (LDH), and endothelial nitric oxide synthase (eNOS), using the following primary antibodies: anti-Mn-SOD 06-984 (Millipore, Milan, Italy), anti-GPx1 ab22604 (Abcam, USA), anti-LDH (H-160) sc-33781 (Santa Cruz Biotec, USA), and anti-eNOS sc-654 (Santa Cruz Biotec, USA). For all assays goat anti-rabbit horseradish peroxidase (HRP) sc-2004 was used as secondary antibody (Santa Cruz Biotec, USA).

### 2.7. Nitrotyrosine Measurement

In order to measure the nitrotyrosine levels as marker of peroxynitrite formation in the cardiac and muscular tissue homogenates a commercial ELISA kit was used (ab113848, Abcam, Cambridge, USA), according the manufacturer's protocol.

### 2.8. Troponin I and Creatine Phosphokinase Levels

Troponin I (TnI) and creatine phosphokinase (CPK) expression levels were quantified by ELISA tests following the manufacturer's instructions (E01T0524 BlueGene and E02C0388 BlueGene, China) starting from tissue homogenates.

### 2.9. Statistical Analysis

Data values are expressed as mean ± s.e.m. of *n* number of rats for the* in vivo* experiments. Statistical analysis was assessed either by Student's *t*-test when each single group of exercise trained (ET) rats receiving oxygen/ozone mixture was compared with the group of ET rats receiving O_2_ (control) only; one-way ANOVA followed by Dunnett's test was used when all the experimental groups in each setting were compared together, in order to assess the variance among the groups. A probability *p* value less than 0.05 was considered significant to reject the null hypothesis.

## 3. Results

### 3.1. Effects of Ozone Treatment on Exercise Capacity

**Figure 1:**
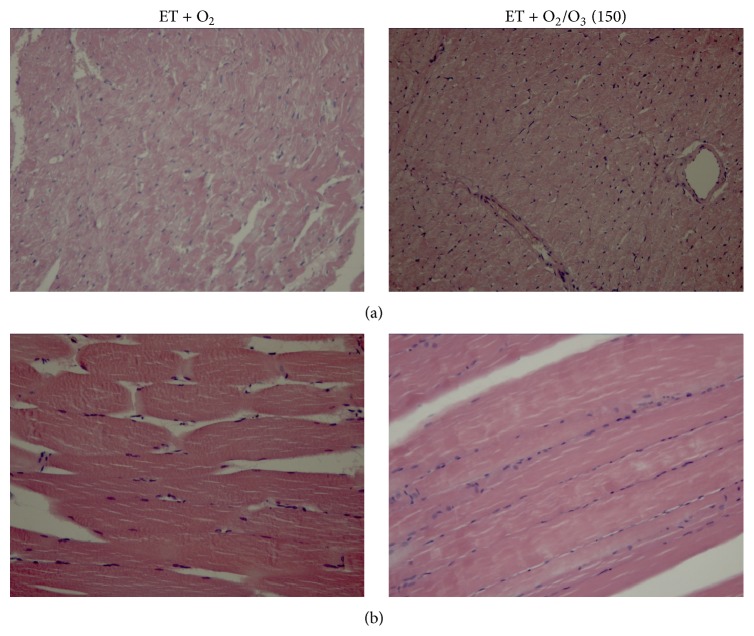
Hematoxylin and eosin staining of cardiac and gastrocnemius tissues from rats subjected to prolonged high intensity exercise and insufflated with oxygen and with 150 *μ*g/kg of oxygen/ozone mixture. Hematoxylin and eosin staining was more evident in heart (a) and gastrocnemius (b) of ET + O_2_ rats compared to rats insufflated with 150 *μ*g/kg of oxygen/ozone mixture, in which the ozone treatment reduced cardiac myocellular and muscular hypertrophy. ET = exercise trained rats running on treadmill.

Rats receiving daily intraperitoneal administration of O_2_ and running on a treadmill covered an average distance of 482.8 ± 21.8 m/week as calculated during all the 5 weeks of treatment (Table 1). This distance was increased in a significant manner in rats receiving daily intraperitoneal administration of an oxygen/O_3_ mixture at a dose of 100; 150; and 300 *μ*g/kg (Table 1). The resistance to the fatigue was dose-dependent and reached its maximum at 300 *μ*g/kg with a 34.5% increase of the distance covered by the rats on the treadmill with respect to the rats receiving O_2_ only. Interestingly, the resistance conferred by oxygen/O_3_ was still and significantly occurs after stopping the administration of oxygen/O_3_. Indeed, the rats that received 150 *μ*g/kg of oxygen/O_3_ showed long running distance even during the following 3 weeks after treatment (Figure 1). 300 *μ*g/kg of oxygen/O_3_ prolonged this effect really for a week more (4th week after treatment) with a significance of *p* < 0.05 with respect to O_2_ control (Figure 1). Although still present during the last week (5th week after treatment), all the effects of the oxygen/O_3_ mixture on the exercise capacity become nonsignificant with respect to the rats receiving O_2_ (Figure 1; *p* > 0.05).

### 3.2. Effects of Ozone Treatment on Physical and Hemodynamic Parameters

Table 2 shows the physical and hemodynamic data obtained at the end of the 5 weeks of oxygen/O_3_ treatment (6th week of the protocol). 150 *μ*g/kg oxygen/O_3_ slightly affected some physical and hemodynamic parameters, but a maximum effect was posed at 300 *μ*g/kg (*p* < 0.01). Moreover, exercised oxygen/O_3_ treated rats (ET + O_2_/O_3_) showed lower MAP (−13%) and HR (−6%) values compared to the exercised ET + O_2_ group; furthermore, ozone treatment reduced of 49% the left ventricular mass (LV mass) and relative wall thickness (RWT) measures. Both systolic and diastolic functions were improved in exercised ET + O_2_/O_3_ rats compared to exercised ET + O_2_ group (Table 2). Finally, oxygen/O_3_ treatment led to higher MPI index with respect to the O_2_ treatment. This effect started from the dose of 150 *μ*g/kg (*p* < 0.05) and was more effective (+14%) at a dose of 300 *μ*g/kg (*p* < 0.01).

All the effects demonstrated by oxygen/O_3_ at end of the 5 weeks of oxygen/O_3_ treatment (6th week of the protocol) were kept almost intact at the end of the 11 weeks of the protocol (Table 3).

### 3.3. Effects of Ozone Treatment on Hypertrophy and Fibrosis

**Figure 2:**
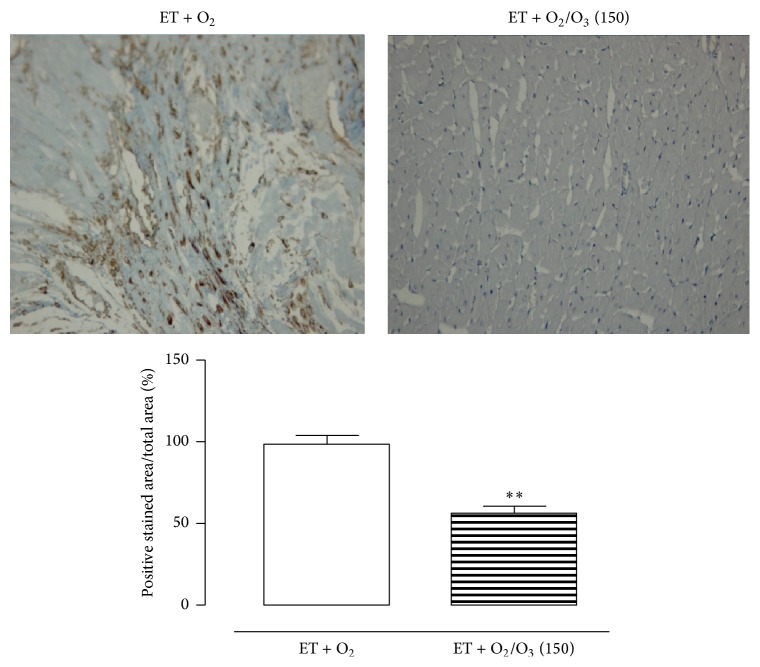
Representative vimentin staining of cardiac tissue from rats subjected to prolonged high intensity exercise and insufflated with oxygen (ET + O_2_) and with 150 *μ*g/kg of oxygen/ozone mixture (ET + O_2_/O_3_). ^*∗∗*^ *p* < 0.01 versus ET + O_2_. ET = exercise trained rats running on treadmill.

**Figure 3:**
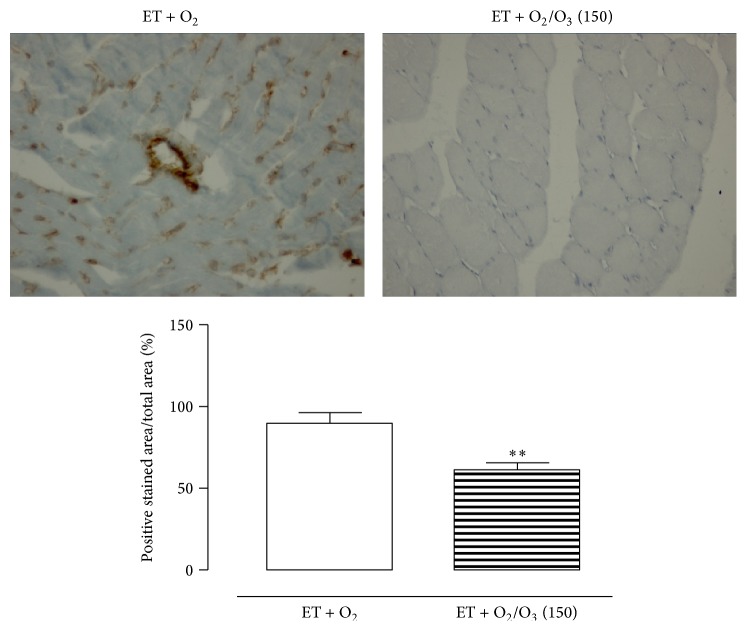
Representative vimentin staining of gastrocnemius tissue from rats subjected to prolonged high intensity exercise and insufflated with oxygen (ET + O_2_) and with 150 *μ*g/kg of oxygen/ozone mixture (ET + O_2_/O_3_). ^*∗∗*^ *p* < 0.01 versus ET + O_2_. ET = exercise trained rats running on treadmill. ET = exercise trained rats running on treadmill.

In Figure 1 are reported the results on gastrocnemius and heart hypertrophy following treatment with the first active oxygen/O_3_ dose of 150 *μ*g/kg. There was an evident muscular and cardiac hypertrophy in exercised ET + O_2_ rats that was reduced after 150 *μ*g/kg i.p. oxygen/O_3_ mixture. Accordingly, Figures 2 and 3 show intense antivimentin labeling in cardiac and muscular tissues of exercised ET + O_2_ rats, indicating stromal fibrosis and presence of fibrocytes that were less evident in cardiac tissue of exercised ET + 150 *μ*g/kg oxygen/O_3_, standing for a reduced perivascular fibrosis.

### 3.4. Effects of Ozone Treatment on Mn-SOD, GPx1, and LDH Expression Levels

**Figure 4:**
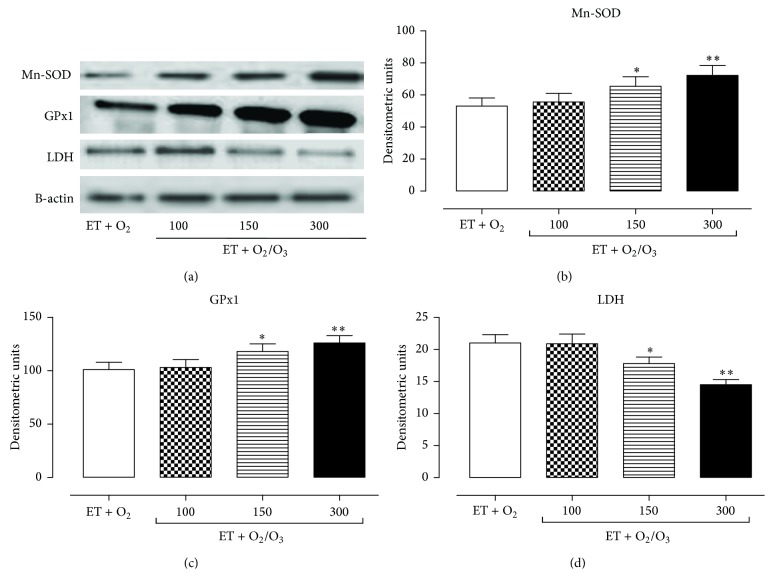
Western blotting analysis of Mn-SOD, GPx1, and LDH expression levels in cardiac tissue. (a) shows the cardiac expression levels of Mn-SOD, GPx1, LDH, and *β*-actin (used as control) in rats subjected to prolonged high intensity exercise and insufflated with oxygen (ET + O_2_) and with oxygen/ozone mixture at three different doses (100, 150, and 300 *μ*g/kg). The expression levels of Mn-SOD (b), GPx1 (c), and LDH (d) are reported as densitometric units (*y*-axis). Starting at dose of 150 *μ*g/kg, with the maximum effect at dose of 300 *μ*g/kg, ozone treatment significantly increased the expression level of the antioxidant enzymes Mn-SOD and GPX, while it lowered the expression levels of LDH. Significant differences with respect to ET + O_2_ are shown as ^*∗∗*^ *p* < 0.05 and ^*∗∗*^ *p* < 0.01. ET = exercise trained rats running on treadmill.

**Figure 5:**
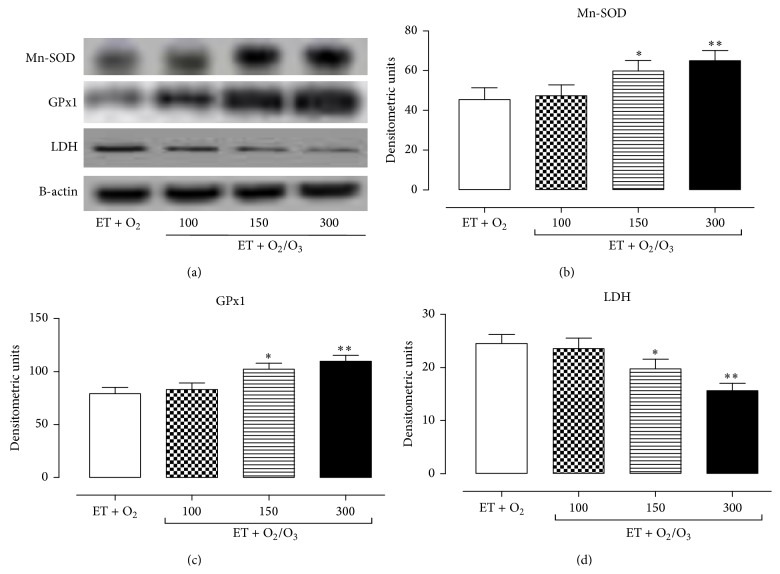
Western blotting analysis of Mn-SOD, GPx1, and LDH expression levels in gastrocnemius tissue. (a) shows the expression levels of Mn-SOD, GPx1, LDH, and *β*-actin (used as control) in the gastrocnemius of trained rats insufflated with oxygen (ET + O_2_) and with oxygen/ozone mixture at three different doses (100, 150, and 300 *μ*g/kg). The expression levels of Mn-SOD (b), GPx1 (c), and LDH (d) are reported as densitometric units (*y*-axis). Starting at dose of 150 *μ*g/kg, with the maximum effect at dose of 300 *μ*g/kg, ozone treatment significantly increased the expression level of Mn-SOD and GPX, while it lowered the expression levels of LDH. Significant differences with respect to ET + O_2_ are shown as ^*∗*^ *p* < 0.05 and ^*∗∗*^ *p* < 0.01. ET = exercised trained rats running on treadmill.

The effects of exercise training and ozone treatment (100, 150, and 300 *μ*g/kg) on Mn-SOD, GPx1, and LDH expression levels were analyzed by Western blotting analysis. In the heart (Figure 4) and gastrocnemius tissues (Figure 5) oxygen/O_3_ treatment (ET + O_2_/O_3_) at the doses of 150 *μ*g/kg (*p* < 0.05) and 300 03BCg/kg (*p* < 0.01) significantly increased the expression levels of the antioxidant enzymes Mn-SOD (a, b) and GPx1 (a, c) compared to the exercised ET + O_2_ rats. Interestingly, the same doses of oxygen/O_3_ mixture resulted in a slight decrease of LDH levels in the heart (Figures 4(a) and 4(d)) and gastrocnemius tissues (Figures 5(a) and 5(d)), showing a beneficial effect of the ozone molecule against the muscular fatigue induced by a prolonged high intensity exercise.

### 3.5. Effects of Oxygen/O_3_ Treatment on eNOS Expression Levels and Peroxynitrite Production

**Figure 6:**
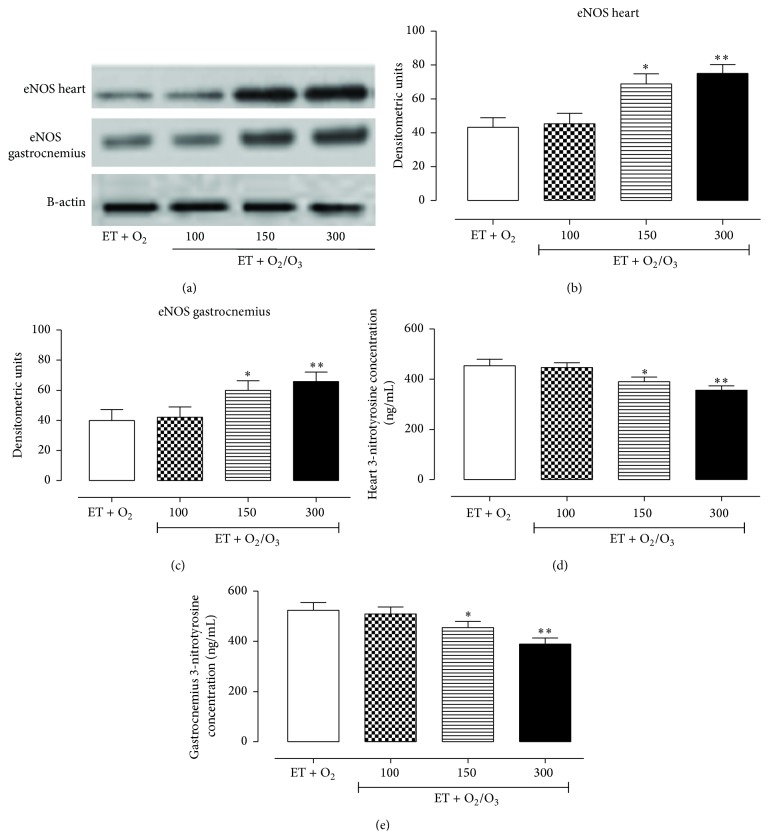
Western blotting analysis of eNOS expression levels and determination of peroxynitrite production in cardiac and gastrocnemius tissues. (a) shows the expression levels of eNOS and *β*-actin (used as control) in heart and gastrocnemius of trained rats insufflated with oxygen (ET + O_2_) and with oxygen/ozone mixture at three different doses (100, 150, and 300 *μ*g/kg). The expression levels of eNOS in heart (b) and gastrocnemius (c) are reported as densitometric units (*y*-axis). (d) and (e) show the 3-nitrotyrosine concentration (ng/mL) (*y*-axis) in all the experimental groups analyzed by ELISA test. At the dose of 150 *μ*g/kg and more effectively at the dose of 300 *μ*g/kg, ozone treatment significantly increased the expression level of eNOS and reduced 3-nitrotyrosine concentration both in cardiac and gastrocnemius tissues. Significant differences with respect to ET + O_2_ are shown as ^*∗*^ *p* < 0.05 and ^*∗∗*^ *p* < 0.01. ET = exercise trained rats running on treadmill.

As shown in Figure 6, a significant increase of eNOS expression levels was evident in exercised ET + O_2_/O_3_ rats both in cardiac (a, b) and muscular tissues (a, c). This started from the dose of 150 *μ*g/kg (*p* < 0.05) and has maximum increase at the dose of 300 *μ*g/kg (*p* < 0.01). In contrast, the oxygen/O_3_ ozone treatment (ET + O_2_/O_3_) reduced the nitrotyrosine concentration with respect to exercised ET + O_2_ rats (d, e).

### 3.6. Effects of Ozone Treatment on CPK and TnI Levels

**Figure 7:**
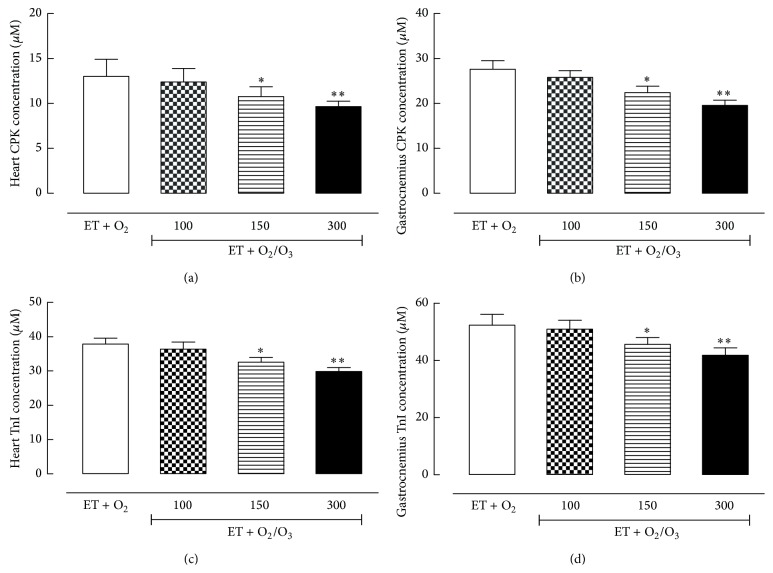
Quantitative determination of creatine phosphokinase (CPK) and troponin I (TnI) levels in cardiac and gastrocnemius tissues. The determination of CPK and TnI concentration (*μ*M) (*y*-axis) has been carried out through ELISA test in heart (a, c) and gastrocnemius tissues (b, d) of all the experimental groups. The levels of CPK and TnI decreased in rats undergoing ozone treatment at the dose of 150 *μ*g/kg (ET + O_2_/O_3_ 150) and more effectively at the dose of 300 *μ*g/kg (ET + O_2_/O_3_ 300) both in cardiac and gastrocnemius tissues. Significant differences with respect to ET + O_2_ are shown as ^*∗*^ *p* < 0.05 and ^*∗∗*^ *p* < 0.01. ET = exercise trained rats running on treadmill.

Both CPK (Figures 7(a) and 7(b)) and TnI (c, d) levels decreased in heart and gastrocnemius tissues of trained rats treated with oxygen/O_3_ mixture at the doses of 150 *μ*g/kg (*p* < 0.05) and 300 *μ*g/kg (*p* < 0.01) compared to the exercised ET + O_2_ rats, underlying an ozone related adaptation to the fatigue.

## 4. Discussion

Ozone therapy has been used in the last three decades as therapeutic agent and potential complement for many disorders [16–19], and several experimental and clinical trials have confirmed its beneficial effects in pathologies related to oxidative and inflammatory burden [14, 20, 21], even though its molecular mechanism has not been completely elucidated. The results presented here point out a new aspect of the ozone therapy, an increased resistance to physical fatigue and stress in rats subjected to high exercise training. They show that during a prolonged intense exercise training the rats insufflated intraperitoneally with oxygen/ozone mixture covered a higher distance per day and per week compared to the oxygen exercise trained rats. Ozone, therefore, increased the resistance of the rats to the physical fatigue and this effect was associated with modifications of physical parameters analyzed at the end of the 11 weeks of protocol. Oxygen/O_3_ treatment lowered cardiac hypertrophy, and the hypertrophy in the gastrocnemius muscle is in accordance with the recent literature [21]. Also, the perivascular fibrosis in the heart and gastrocnemius was less evident in the rats trained under oxygen/O_3_ and prolonged high intensity exercise training. Consequently, LV mass and RWT as well as hemodynamic parameters evaluated at the end of the exercise training period led to beneficial effects. In another literature, the combination of prolonged high intensity exercise training and ozone treatment led to an evident increase of both systolic and diastolic functions and then of the myocardial performance index (MPI), confirming the important role of ozone in the cardioprotection already seen in other settings [14].

Recently, several studies highlighted that high intensity exercise training can induce an increased state of oxidative stress, indicated by a higher presence of oxidized molecules in different tissues that lead to dysfunction and derangement of those tissues [22]. Here, further analysis aimed at evaluating an eventual modulation due to ozone treatment in the levels of oxidative and nitrosative stress showed that two antioxidant enzymes, Mn-SOD and GPx1, were significantly increased after the combination of oxygen/ozone with exercise training. This latter effect supported by the newly described hormetic action of oxygen/ozone [23]. Hormesis is a new and interesting concept of therapy based on the possibility that exposure of cells to a low-stress stimulus produces on these cells a resistance to the stimulus received by promoting the increased activity of endogenous antioxidant mediators such as the superoxide dismutase (SOD) and heme-oxygenase-1 (HO-1) [23]. Such ozone-linked type of action, together with the biomolecular mechanisms activated by low-dose ozone induced “mild stress,” being able in itself to trigger the transcription of protective mediators, may have produced protection from fatigue. Also, oxygen/ozone increased the expression of eNOS both in cardiac and muscular tissues and so the physiological NO bioavailability.

Noteworthy, eNOS generates low levels of nitric oxide (NO) in tissues under normal conditions aimed at maintenance of tissues homeostasis [24]. NO can exert either beneficial or pathological effects, dictated by the amount of NO produced, subcellular localization, and protein interactions of NOS, in addition to the redox state of the cellular environment [25, 26]. Indeed, alterations in NO production from iNOS induction contribute to the pathogenesis of muscular and tissue disorders [27]. These are accompanied by inflammation, resulting in a general increase in NO production which, under conditions of oxidative stress, can lead to generation of peroxynitrite and protein nitration, DNA damage, and apoptosis [28, 29]. Moreover, it is generally held that oxidative stress and inflammation lead to reduced bioavailability of eNOS-derived NO and eNOS “uncoupling,” accompanied by induction of endothelial iNOS expression, which is the source of high, uncontrolled NO that further exacerbates diseases process [30]. Interestingly, our previous studies demonstrated that tissue and damages associated with ischemia/reperfusion infarct can be counteracted by acute ozone pretreatment: a 300 *μ*g/kg oxygen/O_3_ mixture injected intraperitoneally 1 h prior to I/R in Sprague Dawley rats resulted in a significant 2 h cardioprotection and in a decrease of both nitrosative stress and inflammatory and immune responses [14]. Moreover, Borrelli and Bocci [13] reported that oxygen/ozone therapy performed with ozonated autohemotherapy could have an important role in correcting muscle hypoxia, immune dysregulation, and chronic oxidative stress.

Finally, to confirm the role of ozone treatment in reducing muscular fatigue, we analyzed the expression levels of tissue damage biomarkers as LDH, CPK, and TnI in heart and in gastrocnemius tissues. Ozone decreases the LDH, CPK, and TnI levels compared to the untreated trained rats, showing a beneficial effects of this molecule on the tissue damage and on the physical performance.

In conclusion, daily i.p. ozone treatment reduces the muscular fatigue and improves cardiac performance in rats subjected to prolonged high intensity exercise. This was exerted through improvement of NO bioavailability and reduction of oxidative stress to gastrocnemius muscle and to the heart.

## Figures and Tables

**Table 1:** Distance covered during 5 weeks of oxygen/ozone (O_2_/O_3_) treatment (2–6th week of the protocol) by rats subjected to a prolonged high intensity exercise.

	Week 2	Week 3	Week 4	Week 5	Week 6	Weeks 2–6
Mean ± s.e.m. (m/week)						
ET + O_2_	413 ± 23	456 ± 32	486 ± 12	517 ± 32	542 ± 10	482.8 ± 21.8
ET + O_2_/O_3 _100	462 ± 22	503 ± 21	521 ± 21	563 ± 13	587 ± 41	527.2 ± 23.6
ET + O_2_/O_3_ 150	513 ± 31^*∗*^	549 ± 17^*∗*^	559 ± 29^*∗*^	611 ± 28^*∗*^	639 ± 42^*∗*^	574.2 ± 29.4^*∗*^
ET + O_2_/O_3_ 300	559 ± 19^*∗∗*^	601 ± 28^*∗∗*^	641 ± 43^*∗∗*^	708 ± 24^*∗∗*^	735 ± 30^*∗∗*^	648.8 ± 28.8^*∗∗*^

The table shows the mean ± s.e.m. of the distance covered per single week and for the period of 5 weeks (2–6th week of the protocol) application of an oxygen/O_3 _mixture to the four experimental groups of trained rats. Ozone treatment at dose of 150 *μ*g/kg significantly increased (*p* < 0.05) the distance covered compared to the control exercised ET + O_2_ rats. This effect was more evident at the dose of 300 *μ*g/kg, with a significance of *p* < 0.01 versus ET + O_2 _at the same week. ET = exercise trained rats running on treadmill. Significant differences with respect to ET + O_2_ are shown as ^*∗*^ *p* < 0.05 and ^*∗∗*^ *p* < 0.01.

**Table 2:** Physical, hemodynamic, and morphometric parameters recorded after 5 weeks of oxygen/ozone (O_2_/O_3_) treatment (2–6th week of the protocol) in rats subjected to a prolonged high intensity exercise.

	ET + O_2_	ET + O_2_/O_3_ 100	ET + O_2_/O_3_ 150	ET + O_2_/O_3_ 300
Physical parameters				
Body weight (g)	216 ± 28.5	213.2 ± 22.7	200.7 ± 23.7	188.6 ± 19.1
Heart weight (g)	0.81 ± 0.09	0.75 ± 0.10	0.73 ± 0.11	0.71 ± 0.07
Tibial length (cm)	3.89 ± 0.03	3.93 ± 0.04	3.96 ± 0.04	4 ± 0.05
Heart weight/tibial length (g/cm)	0.21 ± 0.06	0.19 ± 0.07	0.18 ± 0.08	0.18 ± 0.05

Hemodynamic parameters				
MAP (mmHg)	129 ± 1.5	128.3 ± 1.7	123 ± 1.4^*∗*^	112 ± 1.1^*∗∗*^
Heart rate (bpm)	431 ± 6.8	427 ± 7.9	415 ± 6^*∗*^	405 ± 5.5^*∗∗*^

Morphometric parameters				
LV mass (g)	0.45 ± 0.07	0.31 ± 0.08	0.28 ± 0.05^*∗*^	0.23 ± 0.03^*∗∗*^
RWT	0.041 ± 0.004	0.043 ± 0.004	0.031 ± 0.002^*∗*^	0.026 ± 0.003^*∗∗*^

Systolic function				
LVFS (%)	51 ± 1.4	51.8 ± 1.2	54.6 ± 1.3^*∗*^	57.2 ± 1.2^*∗∗*^
LVEF %	60.2 ± 1.4	61 ± 1.8	64.2 ± 1.6^*∗*^	66.1 ± 1.5^*∗∗*^
VCF (circ./sec)	0.0065 ± 0.0005	0.0068 ± 0.0003	0.0075 ± 0.0002^*∗*^	0.0082 ± 0.0003^*∗∗*^

Diastolic function				
IVRT (ms)	31 ± 2.5	33.5 ± 3.1	36.6 ± 1.7^*∗*^	39.5 ± 1.6^*∗∗*^
*E*/*A* ratio (ms)	2.9 ± 0.05	2.9 ± 0.06	3.06 ± 0.04^*∗*^	3.1 ± 0.04^*∗∗*^

Global index				
MPI	0.78 ± 0.04	0.79 ± 0.03	0.88 ± 0.03^*∗*^	0.89 ± 0.02^*∗∗*^

MAP = mean arterial pressure; LV = left ventricular mass and relative wall thickness (RWT); EF = ejection fraction; FS = fractional shortening and velocity of circumferential fiber shortening (VCF); IVRT = Absolute left ventricular isovolumetric relaxation time; *E*/*A* ratio = ratio of maximal early diastolic peak velocity/late peak velocity of mitral flow. MPI = myocardial performance index; ms = milliseconds. Significant differences with respect to ET + O_2_ are shown as ^*∗*^ *p* < 0.05 and ^*∗∗*^ *p* < 0.01. ET = exercise trained rats running on treadmill.

**Table 3:** Physical, hemodynamic, and morphometric parameters recorded at the end of the 11 weeks of protocol in rats subjected to a prolonged high intensity exercise.

	ET + O_2_	ET + O_2_/O_3_ 100	ET + O_2_/O_3_ 150	ET + O_2_/O_3_ 300
Physical parameters				
Body weight (g)	272 ± 18.5	271 ± 14.8	268.7 ± 14.4	260 ± 15.1
Heart wt (g)	0.86 ± 0.11	0.79 ± 0.09	0.75 ± 0.08	0.735 ± 0.07
Tibial length (cm)	3.9 ± 0.04	3.93 ± 0.03	3.98 ± 0.02	4.1 ± 0.03
Heart weight/tibial length (g/cm)	0.22 ± 0.04	0.20 ± 0.04	0.19 ± 0.02	0.18 ± 0.02

Hemodynamic parameters				
MAP (mmHg)	125 ± 1.3	123.3 ± 1.1	119 ± 1.5^*∗*^	111 ± 1.2^*∗∗*^
Heart rate (bpm)	436 ± 7.4	431 ± 7.3	413 ± 7.5^*∗*^	403 ± 7.9^*∗∗*^

Morphometric parameters				
LV mass (g)	0.49 ± 0.07	0.35 ± 0.08	0.30 ± 0.05^*∗*^	0.28 ± 0.03^*∗∗*^
RWT	0.045 ± 0.004	0.045 ± 0.004	0.036 ± 0.003^*∗*^	0.031 ± 0.002^*∗∗*^

Systolic function				
LVFS (%)	49 ± 1.1	49.6 ± 1.4	53.7 ± 1.5^*∗*^	53.8 ± 1^*∗∗*^
LVEF %	58 ± 1	59 ± 2.5	62 ± 1.6^*∗*^	64 ± 1.5^*∗∗*^
VCF (circ./sec)	0.0061 ± 0.0003	0.0062 ± 0.0002	0.0070 ± 0.0002^*∗*^	0.0074 ± 0.0003^*∗∗*^

Diastolic function				
IVRT (ms)	29 ± 2.8	29.5 ± 2	34.5 ± 1.2^*∗*^	39 ± 1.8^*∗∗*^
*E*/*A* ratio (ms)	2.4 ± 0.06	2.42 ± 0.05	2.54 ± 0.04^*∗*^	2.63 ± 0.05^*∗∗*^

Global index				
MPI	0.74 ± 0.03	0.75 ± 0.06	0.81 ± 0.02^*∗*^	0.85 ± 0.02^*∗∗*^

MAP = mean arterial pressure; LV = left ventricular mass and relative wall thickness (RWT); EF = ejection fraction; FS = fractional shortening and velocity of circumferential fiber shortening (VCF); IVRT = absolute left ventricular isovolumetric relaxation time; *E*/*A* ratio = ratio of maximal early diastolic peak velocity/late peak velocity of mitral flow. MPI = myocardial performance index; ms = milliseconds. Significant differences with respect to ET + O_2_ are shown as ^*∗*^ *p* < 0.05 and ^*∗∗*^ *p* < 0.01. ET = exercise trained rats running on treadmill; ET + O_2_/O_3_ 100 = rats receiving exercise + 100 *μ*g/kg oxygen/O_3_; ET + O_2_/O_3_ 150 = rats receiving exercise + 150 *μ*g/kg oxygen/O_3_; ET + O_2_/O_3_ 300 = rats receiving exercise + 300 *μ*g/kg oxygen/O_3_.
